# KRAS status predicted by pretreatment MRI radiomics was associated with lung metastasis in locally advanced rectal cancer patients

**DOI:** 10.1186/s12880-023-01173-5

**Published:** 2023-12-12

**Authors:** Yirong Xiang, Shuai Li, Maxiaowei Song, Hongzhi Wang, Ke Hu, Fengwei Wang, Zhi Wang, Zhiyong Niu, Jin Liu, Yong Cai, Yongheng Li, Xianggao Zhu, Jianhao Geng, Yangzi Zhang, Huajing Teng, Weihu Wang

**Affiliations:** 1 Key Laboratory of Carcinogenesis and Translational Research (Ministry of Education/Beijing), Department of Radiation Oncology, Peking University Cancer Hospital and Institute, Beijing, 100142 China; 2 Department of Radiation Oncology, Peking Union Medical College Hospital, Chinese Academy of Medical Sciences & Peking Union Medical College, Beijing, China; 3 Department of Oncology, Tianjin Union Medical Center, Tianjin, China; 4 Blot Info & Tech (Beijing) Co. Ltd, Beijing, China

**Keywords:** Locally advanced rectal cancer, KRAS, Radiomics, Lung Metastasis

## Abstract

**Background:**

Mutated KRAS may indicate an invasive nature and predict prognosis in locally advanced rectal cancer (LARC). We aimed to establish a radiomic model using pretreatment T2W MRIs to predict KRAS status and explore the association between the KRAS status or model predictions and lung metastasis.

**Methods:**

In this retrospective multicentre study, LARC patients from two institutions between January 2012 and January 2019 were randomly divided into training and testing cohorts. Least absolute shrinkage and selection operator (LASSO) regression and the support vector machine (SVM) classifier were utilized to select significant radiomic features and establish a prediction model, which was validated by radiomic score distribution and decision curve analysis. The association between the model stratification and lung metastasis was investigated by Cox regression and Kaplan‒Meier survival analysis; the results were compared by the log-rank test.

**Results:**

Overall, 103 patients were enrolled (73 and 30 in the training and testing cohorts, respectively). The median follow-up was 38.1 months (interquartile range: 26.9, 49.4). The radiomic model had an area under the curve (AUC) of 0.983 in the training cohort and 0.814 in the testing cohort. Using a cut-off of 0.679 defined by the receiver operating characteristic (ROC) curve, patients with a high radiomic score (RS) had a higher risk for lung metastasis (HR 3.565, 95% CI 1.337, 9.505, *p* = 0.011), showing similar predictive performances for the mutant and wild-type KRAS groups (HR 3.225, 95% CI 1.249, 8.323, *p* = 0.016, IDI: 1.08%, *p* = 0.687; NRI 2.23%, *p* = 0.766).

**Conclusions:**

We established and validated a radiomic model for predicting KRAS status in LARC. Patients with high RS experienced more lung metastases. The model could noninvasively detect KRAS status and may help individualize clinical decision-making.

**Supplementary Information:**

The online version contains supplementary material available at 10.1186/s12880-023-01173-5.

## Background

The standard treatment for locally advanced rectal cancer (LARC) is neoadjuvant treatment followed by total mesorectal excision (TME) [1]. However, the strong heterogeneities among individual LARC patients regarding genetic and molecular biology characteristics lead to different treatment responses. Investigating the intrinsic properties and identifying patient subpopulations is of great significance to develop individualized treatment strategies.

Kirsten rat sarcoma (KRAS) mutation, occurring in 30–40% of colorectal cancers [2], has been reported to play a potential role in colorectal cancer tumour development [3]. Previous clinical trials revealed that KRAS is a negative prognostic biomarker for epidermal growth factor receptor (EGFR) inhibitors [4–6]. Despite the inconsistency in its role in predicting neoadjuvant chemoradiotherapy responses [7], recent studies found that KRAS mutation corresponded to a more aggressive subtype and was associated with poor prognosis [8, 9]. In addition, it has been reported that KRAS mutation may be related to distant recurrence in rectal cancer [10, 11]. These previous studies suggested the important role for KRAS status in rectal cancer.

Invasive pathological examination is usually conducted to detect KRAS status. However, sufficient and high-quality tumour tissue samples for KRAS detection may be difficult to obtain due to poor tolerance for invasive procedures and intratumoral heterogeneity. To reduce invasive procedures and avoid repeated sampling, several trials have aroused interest in assessing KRAS status through noninvasive strategies. Radiomics, which converts imaging information to quantitative features, has been developing rapidly in recent years to improve diagnosis, identify genotypes and predict prognosis in rectal cancer [12–14]. Previous studies have revealed that MRI-based radiomics is an effective strategy to predict KRAS mutations [12, 15, 16]. However, most of the previous studies were conducted in a single centre and did not further investigate the association between the model prediction and tumour prognosis. In this multicentre study, we aimed to establish a radiomic model to predict KRAS mutation status in LARC patients. Using this radiomic model, we explored the association between KRAS status and metastasis occurrence after surgery to help individualize treatment decision-making.

## Methods

### Patients

This retrospective multicentre study was conducted at two tertiary academic cancer institutions in China and was approved by the ethics committees of Peking University Cancer Hospital and Institute, the leading site. Participants signed informed consent forms before the study began.

From January 2012 to January 2019, 103 LARC patients were identified. The inclusion criteria were as follows: (1) histologically confirmed rectal adenocarcinoma; (2) stage cT3-4N0M0 or cTxN + M0 (AJCC 8th) confirmed by MRI; (3) standard concurrent neoadjuvant chemoradiotherapy followed by TME; (4) available KRAS mutation status; (5) available MRI images (T2-weighted imaging, T2WI) before neoadjuvant therapy with sufficient image quality; (6) age ≥ 18 years; and (7) Eastern Cooperative Oncology Group (ECOG) score < 2. Eventually, we included 103 LARC patients and randomly divided the patients into training and testing cohorts at a 7:3 ratio, which was 73 and 30 patients, respectively.

The patients’ baseline characteristics were obtained from medical records. The neoadjuvant concurrent chemoradiotherapy strategy was long-course radiation (22–25 fractions of 2–2.3 Gy for primary rectal tumours, with 1.8–2 Gy to the clinical target volume that included the mesorectal area, internal iliac, obturator, and presacral lymphatic drainage area) with concurrent capecitabine 825 mg/m^2^ bid [17]. Tissue samples to determine KRAS status were obtained by preoperative rectal biopsy. KRAS mutations (exons 2, 3 and 4) were identified by real-time polymerase chain reaction (PCR) with an amplification refractory mutation system or next-generation sequencing (NGS). In this cohort, 36 patients were KRAS mutated; while 67 patients were KRAS wild. In the training cohort with 73 LARC patients, KRAS mutation rate was 36.99% (n = 27), while in the testing cohort with 30 LARC patients, the rate was 30% (n = 9).

### Image acquisition and radiomic feature extraction

All patients underwent MRI examinations (3.0-T; Magnetom Skyra, Siemens, Munich, Germany or Signa HDX, GE, Pennsylvania, America) in the supine position within 2 weeks prior to neoadjuvant chemoradiotherapy and no special bowel preparation was performed. Details about MRI parameters from the two centers were listed in supplementary Table 1. The T2W MRI scans in Digital Imaging and Communications in Medicine (DICOM) format were exported and sent to Precision Medicine Open Platform version 2.0.1 (https://www.blothealth.com) [18, 19]. In this software, regions of interest (ROIs) were delineated manually and independently by four board-certified radiation oncologists (> 5 years working experience) and verified by a radiation oncology expert (with > 20 years of experience). To minimize signal heterogeneity, the images were standardized by Z scores. Then, 3D-reconstruction was conducted using the Marching Cube algorithm, and the tumour volume outlined by the ROI was resampled into 1 × 1 × 1-mm voxels. Four groups of radiomics features were extracted, including 48 wavelet features, 42 texture features, 540 histograms of oriented gradient (HOG) features, and 156 statistical features (features definition in Supplementary Table 2).

### Radiomic features selection and model construction

To reduce bias and avoid overfitting, we first removed features with infinite, null or constant values and features with extremely small variance. Then, we calculated the Pearson correlation coefficient between each pair of radiomic features. To eliminate the redundancy, we removed the one with larger mean absolute correlation in pairs with |r| > 0.9. Next, univariate logistic regression analysis was used to investigate the association between each radiomic feature and the patient’s KRAS status. We included the features with *p* < 0.1. Then, 73 radiomic features were selected for further analysis.

The preliminarily selected features were standardized by z score normalization. Then, least absolute shrinkage and selection operator (LASSO) regression was conducted to identify predictive features in the training cohort. Next, the selected key radiomic features were used to construct a radiomic prediction model with the support vector machine (SVM) classifier. Youden index was used to determine cut-off in the training cohort. Then, this cut-off was applied both in the training and testing cohort for risk stratification. The predictive value of the radiomic model and the cut-off was assessed in the training and testing cohort by receiver operating characteristic (ROC) curve analysis, sensitivity, specificity, positive predictive value, negative predictive value, Kappa, F1, balanced accuracy and corrected C-index. The radiomic score distribution was presented, while decision curve analysis (DCA) was conducted to evaluate its net benefits.

### KRAS status and metastasis prognosis

To investigate the prognostic value of KRAS in our cohort, we explored the association of KRAS status with post-treatment metastasis, including metastasis to the lung, liver, bone, abdominal lymph nodes and peritoneum. The diagnosis of lung metastasis was made from thorax contrast-enhanced computed tomography (CT), which was performed at each follow-up. Liver metastasis was detected first by abdominal CT at each follow-up and further diagnosed by abdominal contrast-enhanced CT or liver MRI. Bone metastasis was diagnosed by CT, MRI or positron emission computed tomography, which was performed when the patients developed local symptoms. Abdominal lymph node and peritoneal metastasis were defined on abdominal CT or MRI.

To further explore if a significant association with lung metastasis existed, we conducted Cox regression and Kaplan‒Meier survival analyses, in which significance was detected by the log-rank test. Metastasis-free survival was defined as the time from TME to the first confirmation of metastasis or the last follow-up in patients alive and free from metastasis. The patients were recommended to be followed up every 3 months within the first 2 years after TME, every 6 months within the next 3–5 years and every 1 year thereafter.

### Comparison of the predictive performance of the risk stratification from the radiomic model and KRAS status for lung metastasis

**Fig. 1:**
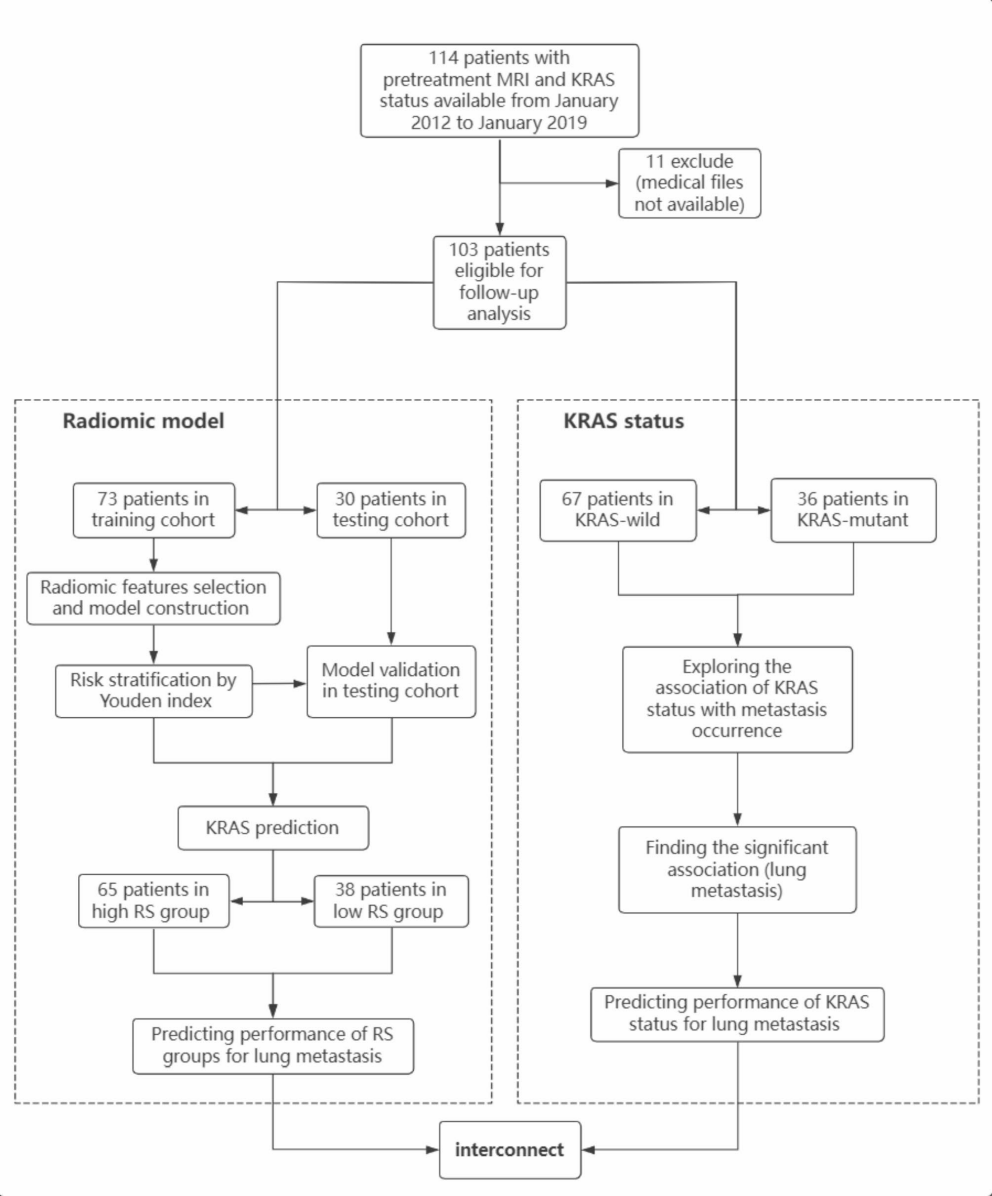
Flow-chart. RS radiomic score

Furthermore, we tested whether the KRAS status predicted and stratified by our radiomic model could represent or substitute the actual KRAS status in predicting metastasis. The association of the risk stratification (high radiomic score group or low radiomic score group) with metastasis occurrence was investigated. Then, whether a significant association with lung metastasis existed was explored by Kaplan‒Meier survival analysis, Cox regression analysis and time-dependent c-index. Finally, the predictive performances of the model stratifying risk or actual KRAS status for lung metastasis were compared by integrated discrimination improvement (IDI) and net reclassification index (NRI). The flow chart is shown in Fig. 1.

### Statistical analysis

Continuous variables are presented as the mean (SD) and were compared by independent-samples *t test*, while categorical variables are presented as numbers (percentage) and were compared by the chi-square test or Fisher’s exact test, when appropriate. Differences were considered statistically significant at *p* < 0.05. Cox regression analyses were used to detect the association of KRAS status or model stratification with lung metastasis. All statistical analyses mentioned above were performed in SPSS version 22.0 (IBM, Armonk, NY, USA). The extraction of radiomic features was implemented using the Python (Pycharm). The packages used for extracting radiomic features include pydicom, xml, numpy, scipy, skimage, os, multiprocessing, min, max, sum, int, float, sort, len, range. Radiomic feature selection and model construction were performed in R software (version 4.0.5; https://www.r-project.org. The packages included glmnet, VIM, foreign, rms, dplyr, caret, rmda, e1071, pROC, and survIDINRI. Kaplan‒Meier survival analysis and log-rank analysis were performed in https://hiplot.com.cn).

## Results

### Patient characteristics

We included 103 LARC patients who were divided into a training cohort (n = 73) and a testing cohort (n = 30). Table 1 shows the demographic and clinical characteristics of these patients, with significant differences between the training and testing cohorts. A total of 36.99% (n = 27) of patients in the training cohort and 30% (n = 9) of patients in the testing cohort had KRAS mutations (*p* = 0.499).

**Table 1:** Demographic and clinical characteristics

	Training cohort (n = 73)		Testing cohort (n = 30)		***p*** value
	**n/mean**	**%/SD**	**n/mean**	**%/SD**	
Age (years)	57.18	10.66	55.33	6.33	0.379
Sex					
Female	23	32.39	5	16.67	0.124
Male	50	70.42	25	83.33	
CEA					
＞5 ng/ml	30	42.25	9	30.00	0.292
≤5 ng/ml	43	60.56	21	70.00	
Pathologic differentiation					
High	4	5.63	3	10.00	0.346
Moderate	48	67.61	16	53.33	
Poor	5	7.04	5	16.67	
Location					
≤5 cm	33	46.48	13	43.33	0.348
5–10 cm	39	54.93	15	50.00	
>10 cm	1	1.41	2	6.67	
Clinical T stage					
cT2	3	4.23	0	0.00	0.396
cT3	54	76.06	21	70.00	
cT4	16	22.54	9	30.00	
KRAS					
Wild	46	63.01	21	70.00	0.499
Mutant	27	36.99	9	30.00	
Pathologic T stage					
pT0	5	7.04	5	16.67	0.288
pT1	3	4.23	1	3.33	
pT2	24	33.80	8	26.67	
pT3	41	57.75	15	50.00	
pT4	0	0.00	1	3.33	
Pathologic N stage					
pN0	39	54.93	20	66.67	0.278
pN1	29	40.85	7	23.33	
pN2	5	7.04	3	10.00	

SD: standard deviation; CEA: Carcinoembryonic antigen

### Radiomic feature selection and model construction

**Fig. 2:**
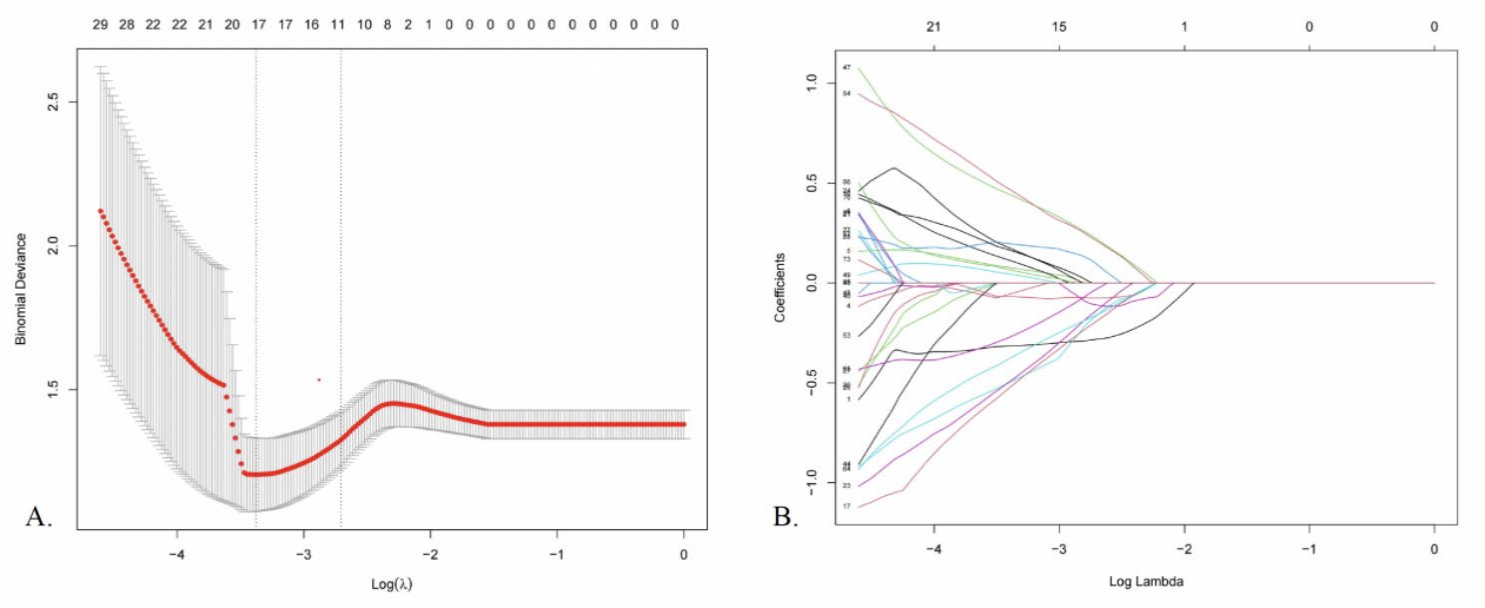
LASSO regression. **(A)** Tenfold cross-validation; two dotted lines represent λmin and λ1se. λmin corresponds to the model with the lowest test error in cross-validation, while λ1se yields a model with moderate complexity, where the error is within one standard error of the minimum error. The optimal λ in this study was calculated by the minimum criteria (λmin). **(B)** LASSO coefficient plot. Each line represents a radiomic feature. LASSO, Least absolute shrinkage and selection operator

**Fig. 3:**
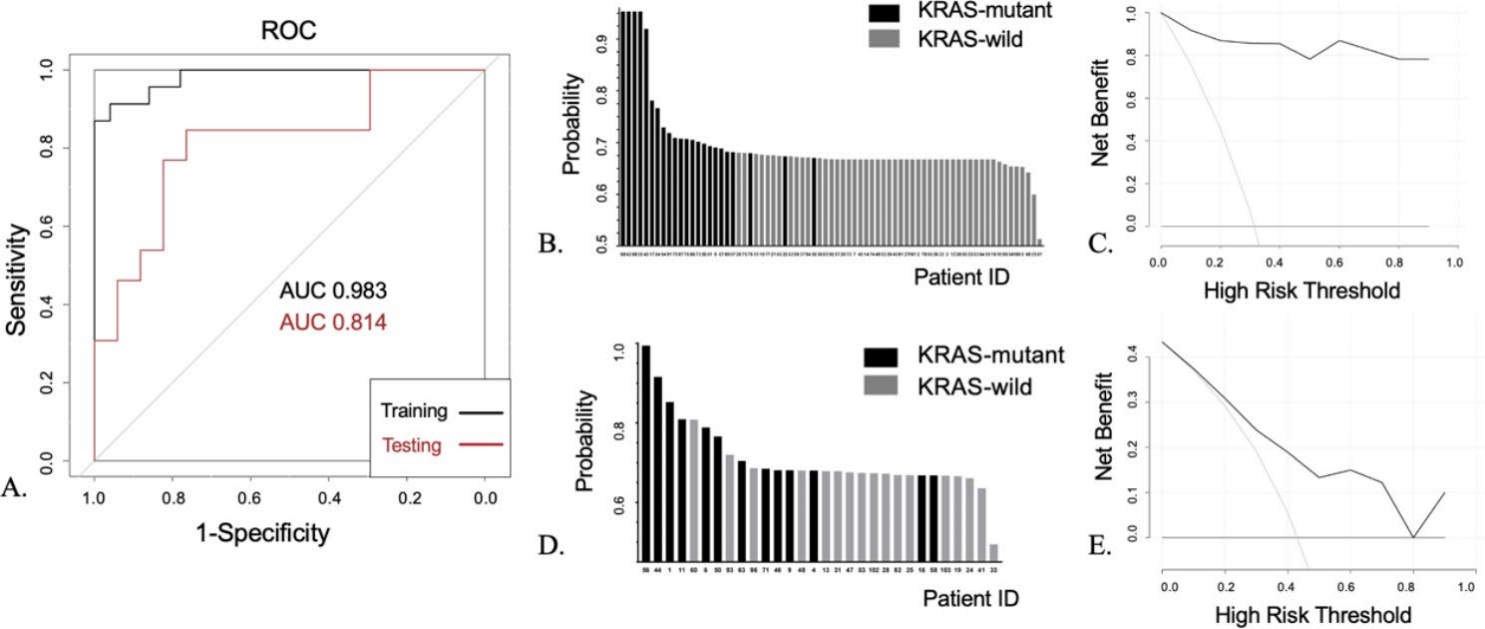
Model performance and validation. **(A)** Receiver operator characteristic (ROC) curve of the radiomics prediction model. Radiomic score distribution in the **(B)** training cohort and **D)** testing cohort. The x-axis represents the patient ID, so each column corresponds to one patient. The y-axis represents the probability of a KRAS mutation predicted by the radiomic model. The black columns indicate that the patients actually had KRAS mutations, while the grey columns indicate that the patients had wild-type KRAS. In the prediction model, a higher column indicates a higher probability for a KRAS mutation. Decision curve analysis (DCA) in the **(C)** training cohort and **E)** testing cohort. The x-axis represents the risk threshold of the radiomic prediction model, while the y-axis represents the net benefits of each threshold. The solid black line represents the radiomic prediction model.

The primary selection by univariate logistic regression analysis identified 73 features for further screening. Tenfold cross-validation for LASSO regression was conducted. **λ**min was applied, leading to the selection of 17 significant features (features listed in Supplementary Table 3). The tenfold cross-validation and coefficients for each feature are shown in Fig. 2. Using these 17 features, we constructed a radiomic prediction model using SVM. The AUCs were 0.983 and 0.814 in the training and testing cohorts, respectively (ROC curve shown in Fig. 3A).

### Model validation

The radiomic prediction distributions are shown in Fig. 3B and D. It was revealed that most patients with KRAS mutations had relatively high radiomic scores. The threshold to discriminate between mutant and wild-type status with the prediction model was determined by the Youden index (sensitivity + specificity-1 = 0.679) in the ROC curve. Decision curve analysis (Fig. 3C and E) indicated that the cut-off value had net benefits in both the training cohort and testing cohort. The prediction performance, as measured by sensitivity, specificity, positive and negative predictive value, Kappa value, F1, balanced accuracy and corrected C-index, is shown in Supplementary Table 4.

### Exploration of the association of KRAS status and risk stratification with metastasis

We first explored the differences in clinical characteristics and prognosis between the KRAS-wild and KRAS-mutant groups (Table 2). There was a statistically significant difference in lung metastasis between these two groups (*p* = 0.010). No significance was found in clinical features or other prognostic outcomes.

**Table 2:** Clinical characteristics and prognosis of the KRAS-wild-type and KRAS-mutant groups

	Wild-type (n = 67)	Mutant (n = 36)	***p***
Age (years, mean, SD)	56.51 (9.62)	56.89 (9.71)	0.849
Sex (n, %)			
Female	17 (25.37)	11 (30.56)	0.573
Male	50 (74.63)	25 (69.44)	-
Pathology (n, %)			
High	5 (7.46)	2 (5.56)	0.916
Moderate	40 (59.70)	24 (66.67)	-
Poor	7 (10.45)	3 (8.33)	-
Not defined	15 (22.39)	7 (19.44)	-
Location (n, %)			
≤5 cm	31 (46.27)	15 (41.67)	0.217
5–10 cm	33 (49.25)	21 (58.33)	-
>10 cm	3 (4.48)	0 (0)	-
Clinical T stage (n, %)			
cT2	1 (1.49)	2 (5.56)	0.510
cT3	50 (74.63)	25 (69.44)	-
cT4	16 (23.88)	9 (25.00)	-
Serum CEA (n, %)			
＞5 ng/ml	45 (67.16)	19 (52.78)	0.151
≤5 ng/ml	22 (32.84)	17 (47.22)	-
Pathologic T stage (n, %)			
pT0	8 (11.94)	2 (5.56)	0.539
pT1	3 (4.48)	1 (2.78)	
pT2	20 (29.85)	11 (30.56)	
pT3	36 (53.73)	21 (58.33)	
pT4	0 (0)	1 (2.78)	
Pathologic N stage (n, %)			
pN0	37 (55.22)	22 (61.11)	0.767
pN1	24 (35.82)	12 (33.33)	
pN2	6 (8.96)	2 (5.56)	
Prognosis			
Lung metastasis (n, %)	7 (10.45)	11 (28.95)	0.010
Liver metastasis (n, %)	5 (7.81)	3 (8.33)	0.875
Bone metastasis (n, %)	4 (5.97)	0 (0)	0.295
Abdominal lymph node or peritoneal metastasis (n, %)	7 (10.45)	1 (2.78)	0.256

CEA: Carcinoembryonic antigen

Using a cut-off of 0.679, the patients were stratified into a high RS group and a low RS group. The clinical features and prognoses of the patients in the high and low RS groups are shown in supplementary Table 5. An association between RS group and lung metastasis was also revealed (9.23% versus 31.58%, *p* = 0.004).

**Fig. 4:**
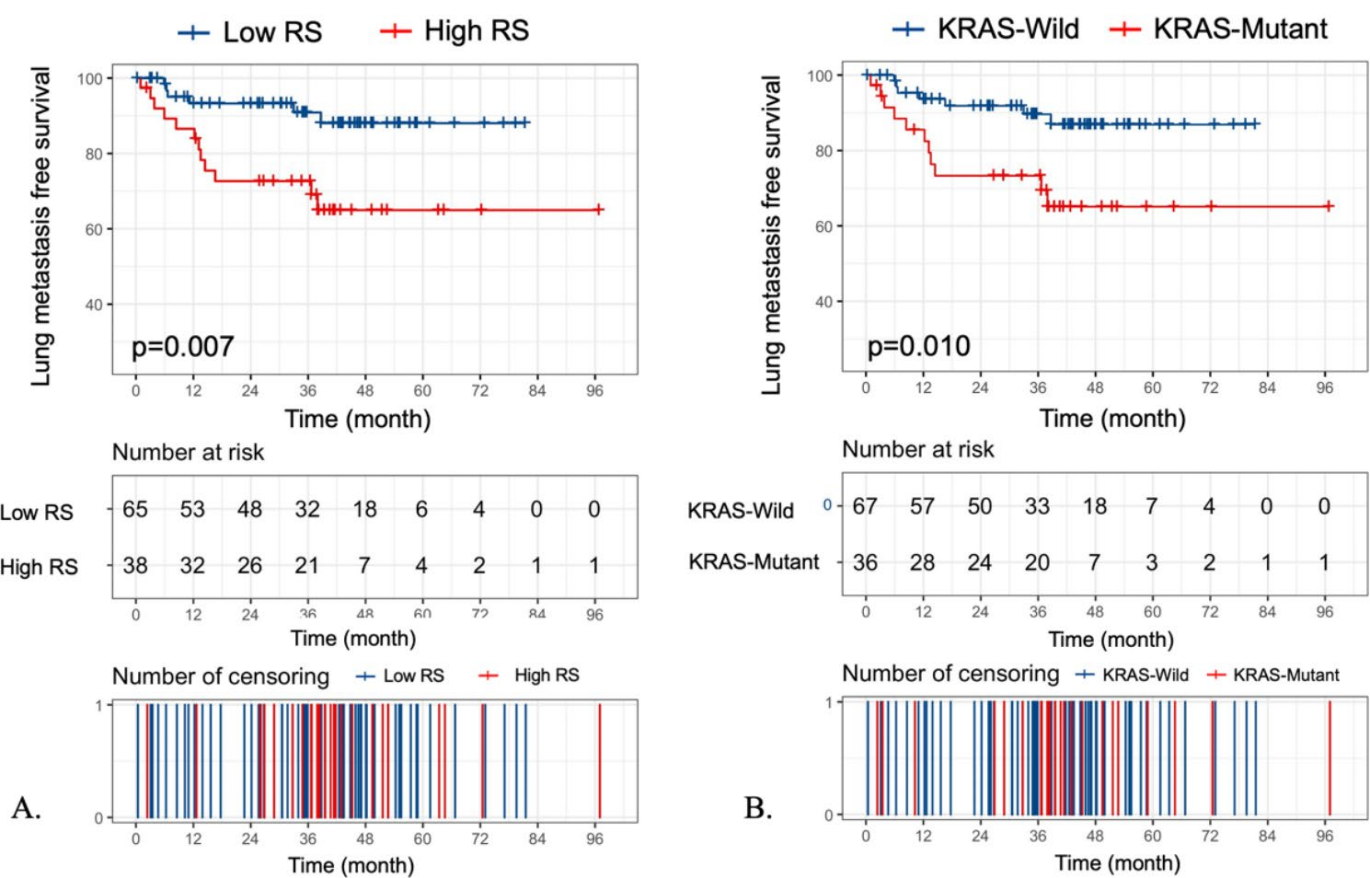
Kaplan–Meier survival curves for patients with lung metastasis in the **(A)** high and low RS score groups; and **(B)** wild-type and mutant KRAS groups. The log-rank test showed significant differences in each comparison (RS group *p* = 0.007; KRAS status *p* = 0.010). RS, radiomic score

We conducted Cox regression to further explore this relationship. The median follow-up duration was 38.1 months (interquartile range: 26.9, 49.4). Seven (10.45%) wild-type KRAS patients developed lung metastasis during follow-up, compared to 11 (28.95%) patients with KRAS mutations (HR 3.225, 95% CI 1.249, 8.323, *p* = 0.016). There were 6 (9.23%) lung metastasis cases in the low RS group, while 11 (31.58%) high RS patients had lung metastasis (HR 3.565, 95% CI 1.337, 9.505, *p* = 0.011). Figure 4 depicts the Kaplan‒Meier survival curves for patients with lung metastasis in the high and low RS groups and in the wild-type and mutant KRAS groups. The lung metastasis-free survival rate was significantly higher in the low RS group than in the high RS group (*p* = 0.007), with the model showing similar predictive performances for the wild-type and mutant KRAS groups (IDI: 1.08%, *p* = 0.687; NRI 2.23%, *p* = 0.766). Time-dependent c-index were depicted in supplementary Fig. 1.

## Discussion

In this study, we developed and validated a radiomic model based on pretreatment MRI scans for predicting KRAS status that had AUCs of 0.983 and 0.814 in the training cohort and testing cohort, respectively. The prediction stratification showed a significant association with lung metastasis.

KRAS is a proto-oncogene involving several tumour-related molecular pathways. Mutant KRAS may lead to constitutive activation of downstream pathways, for example mitogen-activated protein kinase (MAPK) and phosphoinositide-3-kinase/v-akt murine thymoma viral oncogene pathways (PI3K/AKT) [20]. It has been well established that the point mutation status of KRAS is associated with poor response to anti-EGFR treatment [21], and such mutations can even have a detrimental effect on survival [22]. However, the prognostic efficacy of KRAS in LARC patients is still controversial. A recent study included 784 LARC patients from the NCDB database and reported poorer overall survival in KRAS-mutated patients [23]. A population-based analysis indicated that KRAS mutation had prognostic value for poor cancer-specific survival in rectal cancer (HR 1.23, *p* < 0.05) [8]. However, several studies found no associations between KRAS mutation and neoadjuvant chemoradiation response [9, 24] or overall survival [25, 26]. Therefore, although KRAS mutation is relatively common (detected in 30–40% of colorectal cancers [2]), its mechanism in tumour development and invasion in rectal cancer is still unclear. In terms of its association with distant metastasis, few studies have been reported. Margonis et al. recruited 512 colorectal cancer patients who underwent liver metastasis resection. They found that codon 13 KRAS mutation was associated with extrahepatic or lung-specific recurrence rates [11]. Sideris et al. conducted a prospective cohort study and enrolled 135 rectal cancer patients. In this cohort, KRAS mutation was found in 37.4% of patients and was related to distant recurrence in patients with early-stage disease [10]. According to the literature on its molecular mechanism, KRAS activation plays a role in the tumour microenvironment to induce tumorigenesis [27]. That study explored the KRAS-specific effect on triggering tumour cell invasion upon fibroblast stimulation through the HGF-c-MET axis. Additionally, a recent study conducted a transcriptomic analysis and revealed that KRAS-mutated rectal tumour cells could remodel the extracellular matrix around their surrounding fibroblasts [28]. It is believed that mutated KRAS involves the epithelial-mesenchymal transition (EMT) and indicates an invasive nature that helps to facilitate the invasion and metastasis of primary cancer.

Lung is one of the most common metastatic sites in colorectal cancer [29]. Although systemic chemotherapy remains the standard treatment strategy for patients with distant metastasis, recent studies have demonstrated the favourable effect of local treatment for metastasis in terms of long-term survival benefits, especially in patients with “oligometastasis” [30, 31]. Due to their poor condition, combined complications, and multiple metastases, most colorectal patients with lung metastasis are not suitable for surgical resection. In these cases of oligometastases, stereotactic body radiotherapy (SBRT) was shown to be an alternative strategy to local treatment, with improved survival benefits and delayed changes in the systemic therapy regimen [32–34]. Consequently, to achieve better oncologic outcomes, it is important to identify those at risk for oligometastasis and considered appropriate for local treatment in a relatively early stage. KRAS status is an attractive option. It has been reported that colorectal cancer patients with mutated KRAS suffer from more lung metastasis but not liver or peritoneal metastasis [35]. In the present study, we tried to test this association in our cohort. We preliminarily explored the association between KRAS status and metastasis, indicating its potential relationship with lung metastasis with an HR of 3.225. In addition, the risk stratification from the predictive radiomic model showed a similar association with lung metastasis, with an HR of 3.565. From this result, we propose that this noninvasive radiomic model could be applied as a substitute for KRAS status to predict lung metastasis. However, the mechanism about how KRAS status impacts lung metastasis is still unknown, and whether this association exists in other metastases, such as liver or bone metastasis, needs further investigation.

Currently, KRAS mutation detection still meets difficulties in clinical practice. Sufficient tissue sample is needed to conduct next-generation sequencing which is the gold standard approach to detect KRAS mutation status. In the circumstances, postoperative specimens are preferred compared with preoperative tissue sampling biopsy. However, in LARC patients, with the rapid development of neoadjuvant treatment in recent years, more and more patients reached complete or near complete response, leading to less TME surgery to obtain macrodissected sample [36]. Also, the depletion of tumor cellularity after neoadjuvant treatment causes insufficient postoperative tumor samples. So, concordant KRAS mutation status was revealed between pre- and postneoadjuvant therapy [37]. As a result, currently, KRAS mutation is commonly identified by preoperative tissue sampling biopsy in clinical practice. However, due to poor tumor cellularity in sampling biopsy and intratumor heterogeneity, false-negative results pose challenges to pathologists and clinicians in KRAS mutations detection [38]. In addition, intolerance for repeated invasive procedures in tumor patients required alternatively noninvasive methods to determine KRAS status. MRI is a noninvasive and effective tool for diagnosing, staging and evaluating treatment response in rectal cancer. As a bridge between imaging information and personalized treatment [39], MRI-based radiomics has attracted great attention in recent years for its high-throughput extraction of invisible imaging information and transformation of these data into quantitative radiomic features, and its predictive efficacy in rectal cancer is well acknowledged. MRI-based radiomics has been shown to have significant value in improving diagnostic accuracy, assessing treatment response and predicting prognosis [14, 40, 41]. Several previous studies have also depicted the association of MRI radiomics with genetic signatures [42–44]. For KRAS status, Zhang et al. established a T2WI-based radiomic model to predict KRAS status of 82 LARC patients, with C-indexes of 0.801 (95% CI 0.772, 0.830) and 0.703 (95% CI 0.620, 0.786) in the training and validation sets, respectively [15]. In a study with 340 rectal cancer patients, Cui et al. built a predictive model based on T2WI radiomics using SVM classifiers for KRAS mutation with AUCs of 0.722, 0.682, and 0.714 in the training, internal validation and external validation datasets, respectively [12]. Oh et al. found a significant association between a T2WI-based radiomics signature and KRAS mutations [45]. These previous studies indicated that T2W MRI scans could potentially serve as a noninvasive tool to predict KRAS mutation status. In the present study, we used pretreatment T2WI scans to predict KRAS status in LARC patients, with AUCs of 0.983 and 0.814 in the training and testing cohorts, respectively.

The advantages of the present radiomic model lie in three aspects. First, this is a multicentre study, indicating the robustness and practicality of the model. Second, to the best of our knowledge, this is the first exploratory study to associate pretreatment MRI scans to KRAS status and further analyse its association with distant metastasis. We preliminarily investigated the prognostic value of KRAS status for lung metastasis. The model is not only a noninvasive prediction tool for KRAS status but could also be used as a stratification tool for lung metastasis risk. In the future, we would like to further investigate the association of pretreatment MRI radiomic features and lung metastasis in a larger cohort. Third, it is well established that KRAS status indicates the response to anti-EGFR treatment. However, since anti-EGFR treatment is not recommended for LARC patients as neoadjuvant treatment, KRAS status detection is not commonly performed in these patients. In the present study, we offered a noninvasive method to identify KRAS status in LARC patients before neoadjuvant therapy. With increasing evidence of the prognostic value of KRAS status, we hope this study offers a way to obtain some prognostic information in a relatively early stage.

This study also has several limitations. First, the sample size is small. Despite its multicentre natures, LARC patients were not routinely tested for KRAS status. KRAS detection was recommended for patients with simultaneous distant metastasis or those with late stage locally advanced disease and several clinical risk factors. Consequently, a small number of LARC patients were included in the present study, indicating the necessity for further validation in larger, prospective cohorts. Second, we investigated the clinical value of KRAS mutation in risk stratification for lung metastasis. This exploratory study indicated that KRAS may play a role in tumour invasion and metastases and that KRAS status could be a prognostic factor for such events. However, the small sample size and short follow-up duration were two limitations. This association needs to be tested in the future. Third, the two classes of KRAS status were imbalanced. Only 36 patients were KRAS mutant, while in the testing cohort the number is 9. We believe techniques for example Synthetic Minority Over-sampling TEchnique (SMOTE) or stratified cross validation may help overcome this limitation.

## Conclusions

In conclusion, we established and validated a radiomic model for predicting KRAS status using pretreatment T2W MRI scans in LARC patients. The patients in the high RS group suffered from more lung metastases after treatment. This model presents a noninvasive tool to detect KRAS status and may help clinical decision-making.

### Electronic supplementary material

Below is the link to the electronic supplementary material.

Supplementary Material 1

## Data Availability

The datasets used and/or analyzed during the current study are available from the corresponding author on reasonable request.

## Abbreviations

RS: radiomic score
LARC: locally advanced rectal cancer
LASSO: Least absolute shrinkage and selection operator
SVM: support vector machine
AUC: area under the curve
ROC: receiver operating characteristic
SD: standard deviation
CEA: Carcinoembryonic antigen
DCA: Decision curve analysis
