# Supplementary material for: KRAS status predicted by pretreatment MRI radiomics was associated with lung metastasis in locally advanced rectal cancer patients
Source: BMC Med Imaging. 2023 Dec 12;23:210. doi: 10.1186/s12880-023-01173-5 (PMC10717608; doi:10.1186/s12880-023-01173-5)

**Supplementary table 1 T2 weighted MR imaging acquisition parameters.**

|  | Center 1 | Center 2 |
| --- | --- | --- |
| Scanner | SIEMENS 3.0 T (Magnetom Skyra) | GE 3.0 T (Signa HDX) |
| TR(ms) | 4000 | 5160 |
| TE(ms) | 107 | 151 |
| FOV(mm) | 400×400 | 220×220 |
| Flip Angle | 150-175 | 90 |
| Matrix | 640×640 | 320×258 |
| Slice thickness/gap（mm） | 4/0 | 3/0.3 |
| Pixel size（mm） | 0.625×0.625 | 0.688×0.853 |
| Fat suppressed | No | No |

**Abbreviations: TR repetition time, TE echo time, FOV field of view**

**Supplementary table 2 details about radiomic features extracted in the study.**

| Category | Explanation | Numbers |
| --- | --- | --- |
| wavelet features | Capture information about texture and patterns in different frequency bands after wavelet transformation. | 48 |
| texture features | Quantify spatial patterns and variations in pixel intensities within an image, providing information about homogeneity, coarseness, and regularity. | 42 |
| HOG features | Capture the distribution of intensity gradients in different orientations, often used for identifying edges and boundaries. | 540 |
| statistical features | Quantify statistical properties of pixel intensities in an image, including measures such as mean, standard deviation, skewness, and kurtosis. | 156 |

**Supplementary table 3 17 radiomic features selected by LASSO.**

| Features | Category |
| --- | --- |
| Texture.Histogram.Mean | texture features |
| Texture.GLZSM.Large.Zone...Low.Gray.Emphasis |  |
| Wavelet.HLL...Histogram...Kurtosis | wavelet features |
| Hog.0_1_2_1 | HOG features |
| Hog.0_2_2_1 |  |
| Hog.2_0_2_4 |  |
| Hog.2_1_0_5 |  |
| Hog.0_0_1_5 |  |
| Hog.2_1_1_5 |  |
| Hog.2_2_1_11 |  |
| Hog.1_2_0_12 |  |
| Hog.0_2_1_12 |  |
| Hog.2_2_0_14 |  |
| Hog.2_1_1_14 |  |
| Hog.0_1_2_18 |  |
| Hog.0_0_1_19 |  |
| Statistics...1..0..0..Entropy..................... | statistical features |

LASSO, least absolute shrinkage and selection operator; HOG, histograms of oriented gradient

**Supplementary table 4 Prediction performance**

|  | Training cohort | Testing cohort |
| --- | --- | --- |
| Sensitivity | 0.913 | 0.846 |
| Specificity | 0.960 | 0.765 |
| Positive predictive value | 0.913 | 0.733 |
| Negative predictive value | 0.960 | 0.867 |
| Kappa | 0.873 | 0.600 |
| F1 | 0.913 | 0.786 |
| Balanced accuracy | 0.936 | 0.805 |
| C-index | 0.983 | 0.814 |
| Corrected C-index* | 0.985 | 0.815 |

*bootstrapping method, B=1000.

**Supplementary table 5 Clinical** **characteristics and prognoses of the high RS and low RS groups.**

|  | Low RS (n=65) | High RS (n=38) | P value |
| --- | --- | --- | --- |
| Age (years, mean, SD) | 57.08 (9.80) | 55.89 (9.35) | 0.549 |
| Sex (n, %) |  |  |  |
| Female | 17 (26.15) | 11 (28.95) | 0.758 |
| Male | 48 (73.85) | 27 (71.05) | - |
| Pathology (n, %) |  |  |  |
| High | 6 (9.23) | 1 (2.63) | 0.547 |
| Moderate | 38 (58.46) | 26 (68.42) | - |
| Poor | 7 (10.77) | 3 (7.89) | - |
| Not defined | 14 (21.54) | 8 (21.05) | - |
| Location (n, %) |  |  |  |
| <5 cm | 31 (47.69) | 15 (39.47) | 0.238 |
| 5-10 cm | 31 (47.69) | 23 (60.53) | - |
| >10 cm | 3 (4.62) | 0 (0) | - |
| Clinical T stage (n, %) |  |  |  |
| cT2 | 1 (1.54) | 2 (5.26) | 0.351 |
| cT3 | 46 (70.77) | 29 (76.32) | - |
| cT4 | 18 (27.69) | 7 (18.42) | - |
| Serum CEA (n, %) |  |  |  |
| ＞5 ng/ml | 43 (66.15) | 21 (55.26) | 0.272 |
| ≤5 ng/ml | 22 (33.85) | 17 (44.74) | - |
| Pathologic T stage (n, %) |  |  |  |
| pT0 | 8 (12.31) | 2 (5.26) | 0.602 |
| pT1 | 4 (6.15) | 0 (0) |  |
| pT2 | 19 (29.23) | 12 (31.58) |  |
| pT3 | 34 (52.31) | 23 (60.53) |  |
| pT4 | 0 (0) | 1 (2.63) |  |
| Pathologic N stage (n, %) |  |  |  |
| pN0 | 39 (60.00) | 20 (52.63) | 0.450 |
| pN1 | 20 (30.77) | 16 (42.11) |  |
| pN2 | 6 (9.23) | 2 (5.26) |  |
| Prognosis |  |  |  |
| Lung metastasis (n, %) | 6 (9.23) | 12 (31.58) | 0.004 |
| Liver metastasis (n, %) | 4 (6.15) | 4 (10.53) | 0.424 |
| Bone metastasis (n, %) | 4 (6.15) | 0 (0) | 0.119 |
| Abdominal lymph node or peritoneal metastasis (n, %) | 6 (9.23) | 2 (5.26) | 0.468 |

RS, Radiomic score; CEA, Carcinoembryonic antigen

**Supplementary figure 1 Time-dependent C-index plot of radiomic model and KRAS status predicting lung metastasis.**


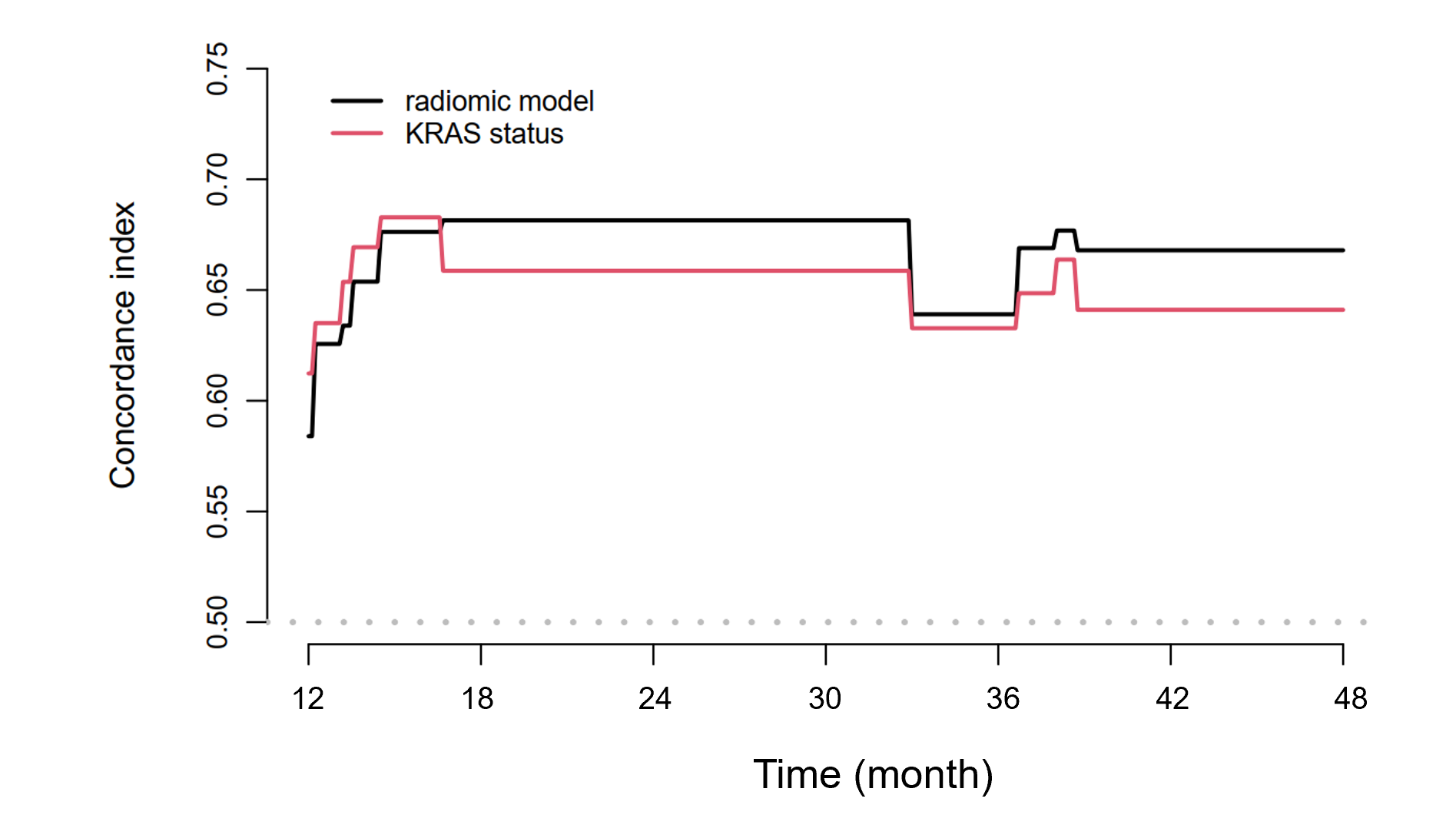

Supplement: Supplementary file 1 — Supplementary Material 1 [file 12880_2023_1173_MOESM1_ESM.docx]
